# Multi-Omics Reveals Carvacrol Inhibits Gas Production in *Pichia manshurica* by Disrupting Membrane Integrity and Energy Metabolism

**DOI:** 10.3390/microorganisms14081615

**Published:** 2026-07-24

**Authors:** Pei Li, Wenqing Wu, Wenmin Pan, Lu Yu

**Affiliations:** 1 Guizhou Key Laboratory of Miao Medicine, Qiandongnan Engineering and Technology Research Center for Comprehensive Utilization of National Medicine, Kaili University, Kaili 556011, China; 18485416587@163.com; 2 School of Liquor and Food Engineering, Guizhou University, Guiyang 550025, China; wuwenqing0809@163.com

**Keywords:** carvacrol, *Pichia manshurica*, gas production inhibition, transcriptome, metabolome

## Abstract

*Pichia manshurica* (*P. manshurica*), a gas-producing spoilage yeast prevalent in fermented foods, causes package swelling, off-flavor formation, and quality deterioration, thereby shortening shelf life and reducing commercial value. Carvacrol, a natural phenolic compound from plant essential oils, has broad-spectrum antimicrobial activity, but its mechanism for inhibiting *P. manshurica*’s gas production is unclear. In this study, in vitro and in situ experiments confirmed that carvacrol significantly inhibits gas production by *P. manshurica* in a concentration-dependent manner. Transcriptomic analysis identified 374 differentially expressed genes (DEGs), which were mainly enriched in biological processes such as nitrogen compound metabolism, lipid metabolism, and organic substance biosynthesis, as well as cellular components including the cell membrane, mitochondrion, and endoplasmic reticulum. Metabolomic analysis screened a total of 440 differentially accumulated metabolites (DAMs), primarily involving carboxylic acids, phospholipids, fatty acids, and amino acids. Integrated transcriptome–metabolome analysis revealed that carvacrol disrupts cell membrane integrity, blocks the tricarboxylic acid cycle and oxidative phosphorylation, and interferes with energy, lipid, and amino acid metabolism in *P. manshurica*, thereby suppressing its gas production. This study elucidates the molecular mechanism by which carvacrol inhibits gas production by *P. manshurica*, providing a theoretical basis for the development and application of carvacrol as a natural preservative in fermented foods.

## 1. Introduction

Fermented foods, including fermented condiments, pickles, sauces, and traditional brewed products, have been indispensable components of the global diet for thousands of years, valued for their unique sensory characteristics, rich nutritional composition, and beneficial microbial metabolites [1]. The complex microbial community involved in fermentation not only shapes the typical flavor and texture of these foods but also contributes to improved digestibility and functional properties [2]. However, microbial spoilage during fermentation, storage, and distribution remains a critical challenge limiting product quality, safety, and shelf life [3,4]. Among spoilage microorganisms, gas-producing yeasts are particularly problematic, as their metabolic activity leads to abnormal gas accumulation, package swelling, product bulging, flavor degradation, and even container rupture, resulting in substantial economic losses and potential food safety risks for both manufacturers and consumers [5].

*Pichia manshurica* (*P. manshurica*) is a prevalent gas-producing yeast frequently isolated from spoiled fermented foods, especially fermented chili sauce, soybean paste, and other high-salt or high-moisture condiments [6,7]. Under favorable conditions, this yeast rapidly proliferates and ferments sugars to produce carbon dioxide and other volatile metabolites, leading to visible spoilage phenomena such as package inflation, unpleasant off-odors, and texture deterioration [8]. Owing to its widespread occurrence and strong spoilage potential, *P. manshurica* has become a major target for microbial control in the fermented food industry [9]. Therefore, developing safe, efficient, and environmentally friendly strategies to inhibit the growth and gas production of *P. manshurica* is urgently needed and of great practical significance for maintaining product quality and extending shelf life.

Traditional synthetic preservatives, such as sodium benzoate, potassium sorbate, and sulfur dioxide, have been widely used to control microbial spoilage in fermented foods [10]. Although these additives exhibit effective antimicrobial activity, their potential toxicity, adverse health effects, and consumer concerns regarding chemical residues have driven increasing demand for natural, clean-label, plant-derived preservatives [11]. Natural phenolic compounds, essential oil components, and plant extracts have attracted extensive attention in recent decades due to their broad-spectrum antimicrobial properties, low toxicity, and good consumer acceptance [12,13]. Among these, carvacrol (2-methyl-5-isopropylphenol), a major active phenolic constituent of oregano, thyme, and other aromatic plant essential oils, has been well documented to exert strong inhibitory effects against a wide range of bacteria and fungi [14]. Previous studies have demonstrated that carvacrol disrupts microbial cell membrane integrity, impairs mitochondrial function, inhibits energy metabolism, and interferes with cell wall synthesis, thereby suppressing microbial growth and reproduction [15,16]. Despite these advances, most existing research has focused on the general antimicrobial effects of carvacrol on pathogenic or spoilage microorganisms, while its specific inhibitory mechanism against gas-producing yeasts, especially *P. manshurica*, remains largely unknown. In particular, how carvacrol interferes with the gas production metabolism of *P. manshurica* at the molecular level has not been systematically explored, which severely limits its rational application and further development as a targeted anti-spoilage agent in fermented foods.

Transcriptomics and metabolomics are powerful, high-throughput omics technologies that enable comprehensive profiling of gene expression and metabolite changes in microorganisms under environmental stress [17,18]. Integrated transcriptome–metabolome analysis can systematically reveal the key genes, metabolites, and regulatory pathways involved in microbial stress responses, providing deep insights into the molecular mechanisms of antimicrobial action [19]. To date, multi-omics approaches have been widely applied to investigate microbial responses to natural preservatives, but few studies have focused on the gas-producing spoilage yeast *P. manshurica* or explored the molecular mechanism underlying carvacrol-mediated inhibition of gas production.

In this study, we performed in vitro and in situ gas production assays to verify the inhibitory effect of carvacrol on *P. manshurica*, and then conducted transcriptome sequencing and untargeted metabolomics analysis to systematically characterize the molecular responses of *P. manshurica* to carvacrol stress.

## 2. Materials and Methods

### 2.1. Strain and Culture Conditions

*P. manshurica* (CQ17) was isolated from spoiled fermented chili sauce in our previous study [6]. The strain was cultured in yeast extract–peptone–dextrose (YPD) medium (1% yeast extract, 2% peptone, and 2% glucose) at 28 °C with shaking at 180 rpm.

### 2.2. In Vitro and In Situ Gas Production Assays

In vitro gas production was measured using a syringe-based system technique described by Vuma et al. [20]. Carvacrol (5 mg) was dissolved in 1 mL of anhydrous ethanol and subsequently diluted with 500 mL of sterile water to prepare a stock solution with a concentration of 10 mg/L. For in vitro validation, this stock solution was added to 20 mL of YPD medium to prepare solutions having concentrations of 0, 0.25, 0.50, 0.75, 1.00, and 1.25 mg/L. *P. manshurica* (40 μL, 10^6^ CFU/g) was cultured in 20 mL of YPD medium within anaerobic bottles equipped with a 2 mL sterile syringe at 28 °C for 12 h. Subsequently, the volume of the gas produced in the sterile syringe was visually inspected and quantified. For in situ validation, this stock solution was added to 20 g of sterilized chili sauce in anaerobic bottles equipped with a 2 mL sterile syringe to prepare samples with carvacrol concentrations of 0, 0.05, and 0.10 g/kg. Then, 40 μL of *P. manshurica* (10^6^ CFU/g) was inoculated and treated with carvacrol at concentrations of 0, 0.05, and 0.10 g/kg. The samples were incubated at 28 °C for 7 days, and the gas production was visually inspected and quantified.

### 2.3. Transcriptome Sequencing

After 12 h of cultivation, the cells of *P. manshurica* (treated by carvacrol at concentration of 0.25 mg/L) from three independent biological replicates were collected by centrifugation (6000× *g*, 5 min, 4 °C). The cells of *P. manshurica* not treated by carvacrol were used as the control group. Total RNA was extracted using the Trizol reagent according to the instructions (Invitrogen, Carlsbad, CA, USA). RNA quality was assessed using a NanoDrop 2000 spectrophotometer (Thermo Fisher Scientific, Waltham, MA, USA) and agarose gel electrophoresis. cDNA libraries were constructed and sequenced on the Illumina HiSeq 2500 platform (Illumina, San Diego, CA, USA). Raw reads were filtered to remove low-quality reads and adapter sequences. Clean reads were mapped to the *P. manshurica* reference genome using HISAT2 [21]. Gene expression levels were quantified using the fragments per kilobase of transcript per million mapped reads (FPKM) method [22]. Differentially expressed genes (DEGs) were identified using DESeq2 with thresholds of log_2_(fold change) ≥ 1 and adjusted *p* < 0.05 [23]. Gene Ontology (GO) and Kyoto Encyclopedia of Genes and Genomes (KEGG) enrichment analyses were performed using clusterProfiler [24]. All transcriptome experiments were performed with three biological replicates to ensure statistical reliability.

### 2.4. Metabolomic Profiling

The cells of *P. manshurica* (treated by carvacrol at concentration of 0.25 mg/L for 12 h) from three independent biological replicates were collected as described above and quenched with liquid nitrogen. The cells of *P. manshurica* not treated by carvacrol were used as the control group. The cells (approximately 100 mg) were extracted using 2 mL of 80% methanol (*v*/*v*) at −20 °C for 30 min. The supernatant was collected by centrifugation (12,000× *g*, 15 min, and 4 °C) and analyzed using ultra-high-performance liquid chromatography coupled with quadrupole time-of-flight mass spectrometry (UPLC-Q-TOF-MS, Agilent Technologies, Santa Clara, CA, USA) [25]. The raw data have been deposited in the MetaboLights database under the accession number MTBLS15055. Metabolite identification was performed by comparing mass spectra and retention times with authentic standards and database searches (HMDB, Metlin) [26]. Peak alignment and quantification were performed using XCMS [27]. Differentially accumulated metabolites (DAMs) were identified with thresholds of variable importance in projection (VIP) ≥ 1 and *p* < 0.05 [28]. KEGG pathway enrichment analysis was conducted to identify key metabolic pathways [24]. The metabolomics analysis was conducted with three biological replicates.

## 3. Results

### 3.1. Effect of Carvacrol on Gas Production of P. manshurica

**Figure 1:**
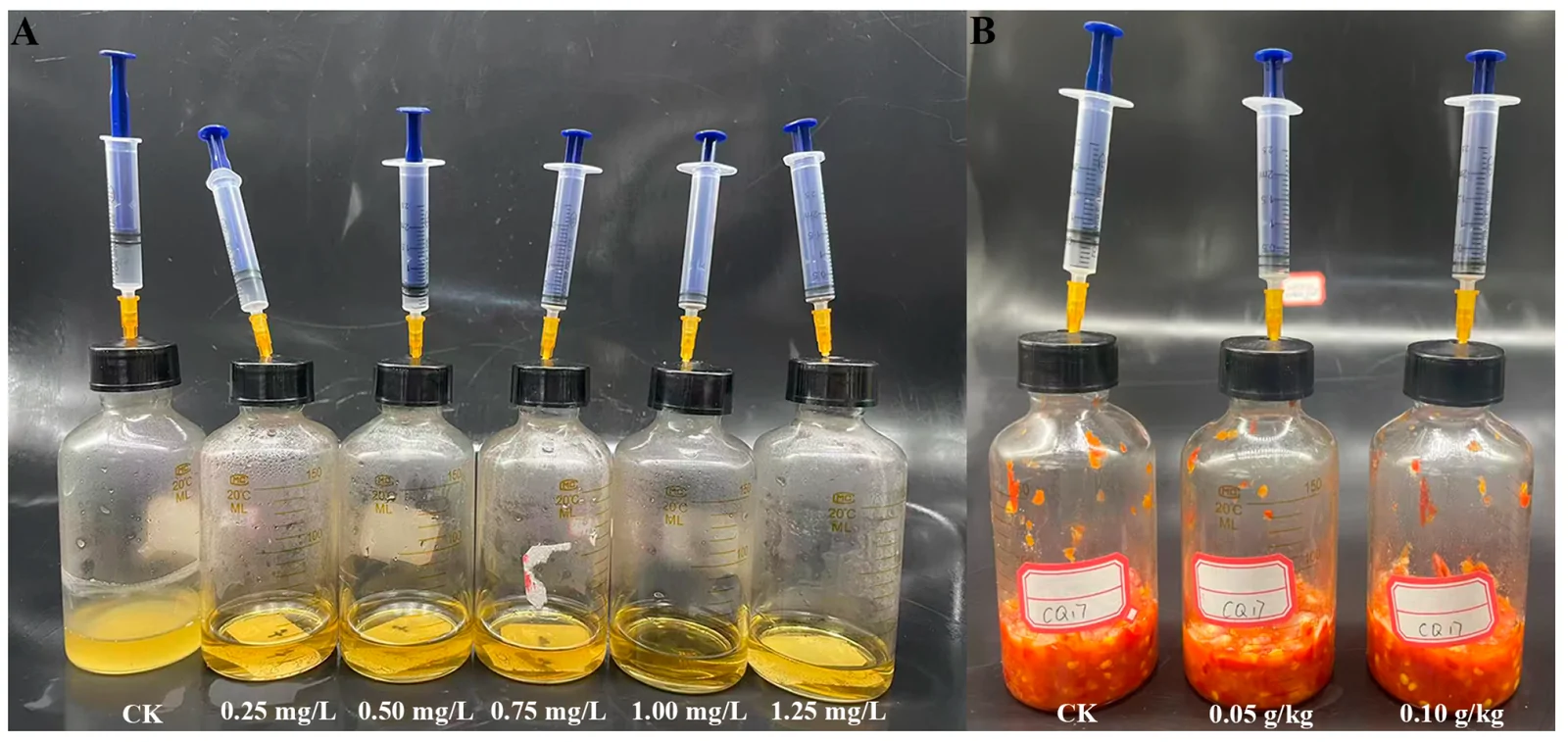
(**A**) Effect of different carvacrol concentrations on gas production by *P. manshurica* in vitro. (**B**) Effect of different carvacrol concentrations on gas production by *P. manshurica* in chili sauce.

As shown in Figure 1A, the in vitro assay revealed that carvacrol not only suppressed yeast growth but also directly targeted the gas-producing phenotype, a previously unreported anti-spoilage mechanism. In contrast to robust gas accumulation in the untreated control (CK group), carvacrol treatment induced a dramatic, dose-dependent reduction in gas volume, with near-complete inhibition achieved at 0.75, 1.00, and 1.25 mg/L (Table 1). This finding challenges the conventional view that carvacrol’s preservative action is limited to general antimicrobial growth inhibition, highlighting its unique capacity to interfere with specific metabolic pathways driving gas formation in spoilage yeasts.

To translate this discovery into practical food applications, we validated the efficacy of carvacrol in a complex, real-world matrix: chili sauce (Figure 1B and Table 1). The results demonstrated that carvacrol at concentrations as low as 0.05 g/kg significantly reduced gas production, while 0.10 g/kg completely eliminated visible signs of spoilage. Critically, this is the first report confirming the effectiveness of carvacrol against *P. manshurica*-mediated gas spoilage in an actual fermented food system. Unlike many studies that rely solely on in vitro data, our in situ validation bridges the gap between laboratory findings and industrial applicability, proving that carvacrol can function as a natural anti-spoilage agent without requiring harsh synthetic additives.

### 3.2. Transcriptomic Responses of P. manshurica to Carvacrol Stress

**Figure 2:**
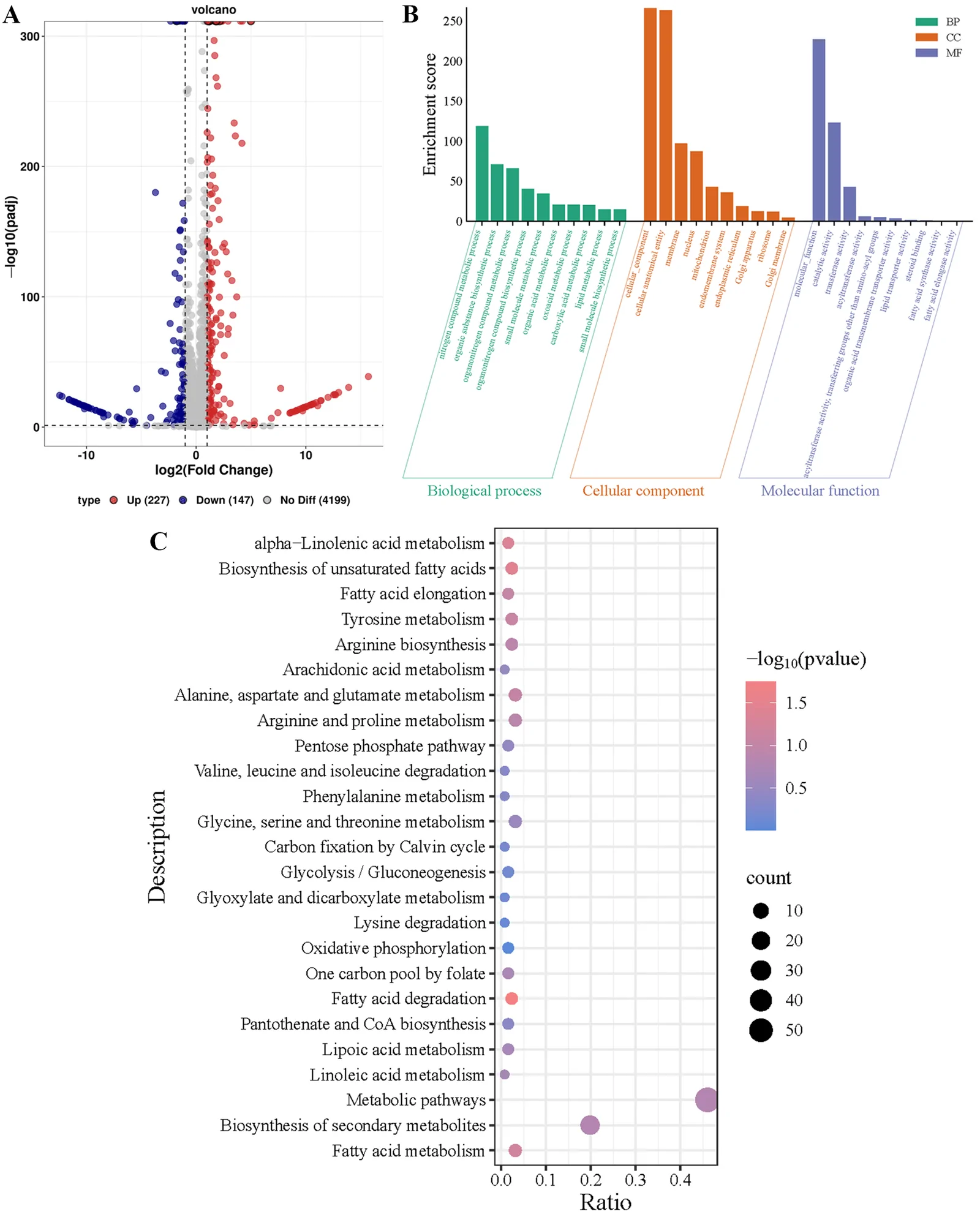
Transcriptomic analysis of *P. manshurica* under carvacrol stress. (**A**) Volcano plot of DEGs. Red dots represent up-regulated genes, blue dots represent down-regulated genes, and gray dots represent non-differentially expressed genes. (**B**) GO enrichment analysis of DEGs. BP: Biological process; CC: Cellular component; MF: Molecular function. The *x*-axis represents the number of DEGs, and the *y*-axis represents the GO terms. (**C**) KEGG pathway enrichment analysis of DEGs. The *x*-axis represents the enrichment ratio, the *y*-axis represents the pathway name, the color of the dots indicates the significance level (−log_10_(*p*-value)), and the size of the dots indicates the number of DEGs enriched in the pathway.

To conduct a systematic investigation into the molecular mechanisms underlying the carvacrol-induced inhibition of gas production in *P. manshurica*, high-coverage transcriptome sequencing was performed. A total of 374 significantly different DEGs were identified, including 227 up-regulated and 147 down-regulated genes, with strict statistical thresholds applied (Figure 2A). The volcano plot revealed clear separation between the CK and treatment groups, with no significant batch effects or low-quality signals observed, confirming the reliability of the transcriptomic data. GO enrichment analysis further validated the consistency of functional perturbations induced by carvacrol (Figure 2B and Table S1). The most significantly enriched biological processes were associated with nitrogen compound metabolism, organic substance biosynthesis, and lipid metabolism, while cellular components were predominantly mapped to the membrane, mitochondrion, and endoplasmic reticulum. These results were highly consistent across replicates, with enrichment scores demonstrating strong statistical confidence, ruling out random variation. Notably, the enrichment of membrane- and mitochondrion-related terms aligned consistently with the known mode of action of carvacrol, providing functional validation of the transcriptomic findings. KEGG pathway enrichment analysis identified core metabolic pathways significantly altered by carvacrol, including fatty acid metabolism, oxidative phosphorylation, amino acid metabolism, and glycolysis/gluconeogenesis (Figure 2C and Table S2). The pathways were enriched with high statistical significance, and the ratio of DEGs to total pathway genes was consistently high, indicating that these pathways were genuinely perturbed rather than being artifacts of multiple testing. Collectively, these transcriptomic data demonstrate that carvacrol induces widespread changes in gene expression in *P. manshurica*, particularly affecting fatty acid metabolism, amino acid metabolism, and central energy metabolism. These findings provide a molecular basis for understanding the antimicrobial mechanism of carvacrol, likely through disruption of membrane lipid homeostasis and interference with core cellular metabolic processes.

### 3.3. Metabolomic Responses of Pichia manshurica to Carvacrol Stress

**Figure 3:**
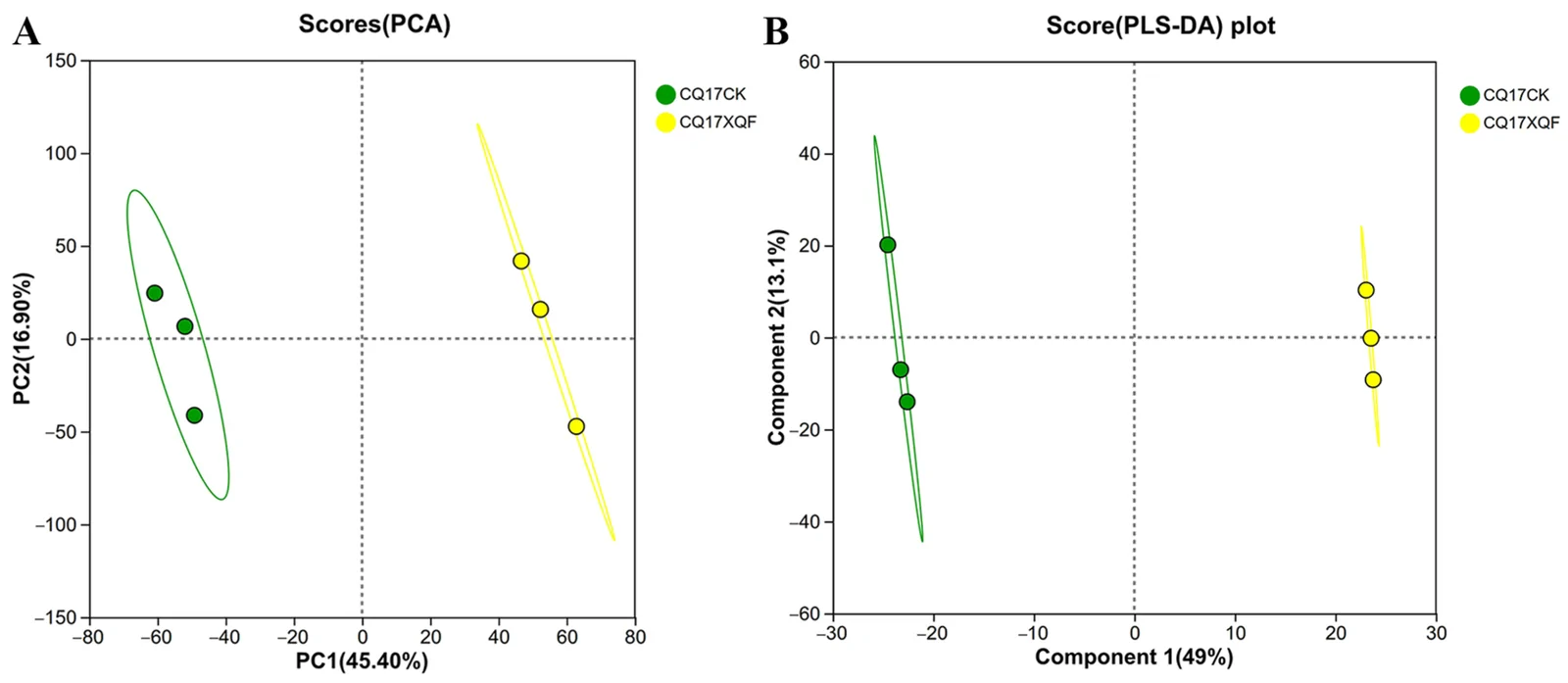
Multivariate statistical analysis of metabolomic data. (**A**) PCA score plot showing separation between the control (CQ17CK, green) and carvacrol-treated (CQ17XQF, yellow) groups. (**B**) PLS-DA score plot confirming clear discrimination between the two groups. The percentages indicate the variance explained by each principal component.

To confirm the reliability and reproducibility of metabolomic responses in *P. manshurica* to carvacrol treatment, we performed two complementary multivariate statistical analyses: unsupervised Principal Component Analysis (PCA) and supervised Partial Least Squares Discriminant Analysis (PLS-DA). The PCA score plot (Figure 3A) revealed clear separation between the control (CQ17CK) and carvacrol-treated (CQ17XQF) groups, with no overlap between the two clusters. The first two principal components explained 45.40% and 16.90% of the total variance, respectively, accounting for over 62% of the metabolic variation. Importantly, the biological replicates within each group were tightly clustered, demonstrating excellent reproducibility and minimal technical variation. The ellipse representing the 95% confidence interval further confirmed that the separation between groups was statistically robust.

Consistent with the PCA results, the PLS-DA score plot (Figure 3B) showed distinct discrimination between the control and treated groups, with Component 1 and Component 2 explaining 49% and 13.1% of the variance, respectively. The tight grouping of replicates within each treatment group, combined with the complete separation of the two clusters, provides strong evidence that the observed metabolic differences are driven by carvacrol treatment rather than batch effects or experimental artifacts. These high-confidence multivariate analyses validate the reliability of our metabolomic dataset. The clear separation between groups, together with the tight clustering of replicates, establishes a solid foundation for downstream identification of differentially accumulated metabolites and key metabolic pathways.

**Figure 4:**
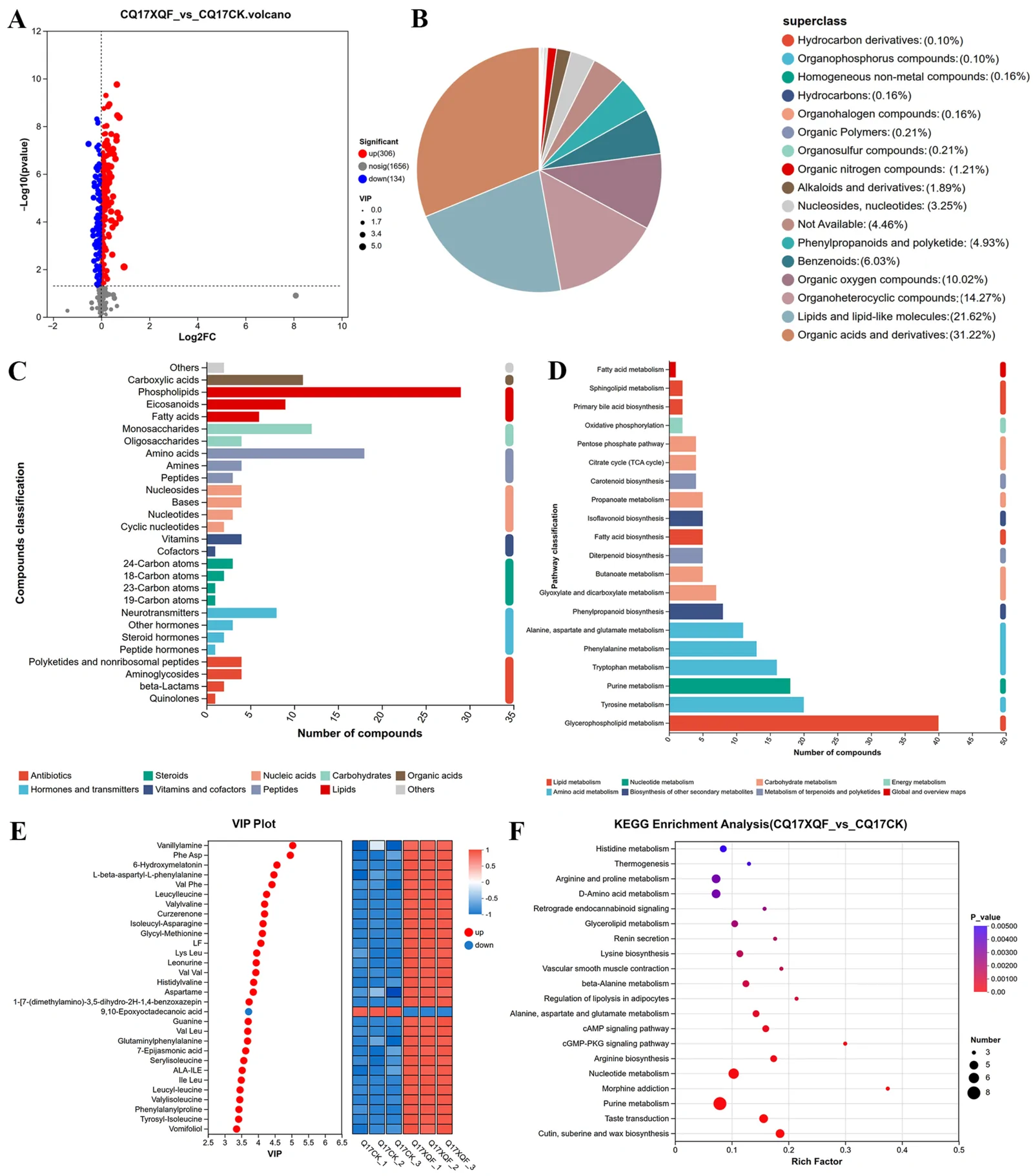
Metabolomic analysis of *P. manshurica* under carvacrol stress. (**A**) Volcano plot of DAMs. Red dots represent significantly up-regulated metabolites, blue dots represent significantly down-regulated metabolites, and gray dots represent non-differentially accumulated metabolites. (**B**) Superclass classification of identified metabolites. The pie chart shows the distribution of metabolites in different superclasses, with the percentage of each category indicated. (**C**) Compound classification of DAMs. The bar chart displays the number of DAMs in each compound class. (**D**) Pathway classification of DAMs. The bar chart shows the number of DAMs involved in different metabolic pathways. (**E**) VIP plot of DAMs. The left panel shows the VIP values of metabolites, and the right panel is a heatmap displaying the relative abundance of the top DAMs in the control (CQ17CK) and carvacrol-treated (CQ17XQF) groups. (**F**) KEGG pathway enrichment analysis of DAMs. The *x*-axis represents the enrichment factor, the *y*-axis represents the pathway name, the color of the dots indicates the significance level (−log_10_(*p*-value)), and the size of the dots indicates the number of DAMs enriched in the pathway.

To further characterize the metabolic responses of *P. manshurica* to carvacrol treatment, we performed untargeted metabolomic analysis, revealing widespread and statistically significant changes in metabolite profiles (Figure 4). Metabolomic profiling of *P. manshurica* under carvacrol treatment revealed widespread metabolic remodeling. As shown in Figure 4A and Table S3, a total of 440 DAMs were identified, including 306 significantly up-regulated and 134 significantly down-regulated metabolites. The distribution of DAMs showed that the majority of significantly altered metabolites were up-regulated, indicating a strong adaptive metabolic response of *P. manshurica* to carvacrol stress. To further characterize the chemical nature of these changes, the identified metabolites were classified by their superclass (Figure 4B and Table S4). The largest proportion of metabolites belonged to organic acids and derivatives (31.22%), followed by lipids and lipid-like molecules (21.62%), organoheterocyclic compounds (14.27%), and organic oxygen compounds (10.02%). The high proportion of organic acids and derivatives reflects major perturbations in central carbon metabolism and the tricarboxylic acid (TCA) cycle, while the abundance of lipid-related metabolites indicates significant alterations in membrane lipid homeostasis. Other notable classes included benzenoids, phenylpropanoids, and nucleotides, suggesting broader impacts on secondary metabolism and nucleic acid turnover.

To further dissect the chemical and functional distribution of the identified DAMs, we performed compound classification and pathway classification analyses. Compound classification (Figure 4C and Table S5) revealed that phospholipids were the most abundant class of DAMs, with more than 30 significantly altered metabolites, followed by amino acids, carboxylic acids, fatty acids, and eicosanoids. The high number of phospholipid and fatty acid-related DAMs indicates profound disruption of lipid metabolism, consistent with transcriptomic evidence of membrane damage. The significant changes in amino acids and carboxylic acids further reflect perturbations in central carbon and nitrogen metabolism, which are critical for energy production and protein synthesis. Pathway classification (Figure 4D and Table S6) provided functional context for these changes. The glycerophospholipid metabolism pathway was the most heavily affected, containing over 40 DAMs, directly supporting the conclusion that carvacrol disrupts membrane lipid homeostasis. Purine metabolism was the second most affected pathway, with approximately 20 DAMs, indicating severe impairment of nucleotide and energy metabolism. Additionally, multiple amino acid metabolism pathways, including tyrosine, tryptophan, and phenylalanine metabolism, showed substantial alterations, further confirming the dysregulation of nitrogen metabolism and protein synthesis. Lipid-related pathways such as fatty acid metabolism and sphingolipid metabolism, as well as energy metabolism pathways including the citrate cycle and oxidative phosphorylation, were also significantly perturbed.

To identify key metabolites contributing to the separation between groups, VIP analysis was performed (Figure 4E and Table S7). A total of 30 metabolites with the highest VIP values (VIP > 3) were selected, most of which were significantly up-regulated in the carvacrol-treated group, as shown in the heatmap. These high-VIP metabolites, including vanillylamine, Phe-Asp, and L-beta-aspartyl-L-phenylalanine, represent potential biomarkers of carvacrol-induced stress in *P. manshurica*. KEGG pathway enrichment analysis further revealed the functional implications of these metabolic changes (Figure 4F and Table S8). The purine metabolism pathway was the most significantly enriched, with the largest number of DAMs and the lowest *p*-value, indicating profound disruption of energy and nucleotide metabolism. Nucleotide metabolism and several amino acid metabolism pathways, including alanine, aspartate, and glutamate metabolism, arginine and proline metabolism, and D-amino acid metabolism, were also highly enriched.

### 3.4. Integrated Transcriptomic and Metabolomic Analysis

Integrated analysis of transcriptomic and metabolomic data revealed a coordinated molecular response of *P. manshurica* to carvacrol stress, with consistent perturbations in lipid metabolism, energy metabolism, and amino acid metabolism at both gene and metabolite levels.

At the transcript level, 374 DEGs were predominantly enriched in membrane-related cellular components, mitochondrial function, and core metabolic processes, including fatty acid metabolism, oxidative phosphorylation, and amino acid metabolism. Metabolomic profiling further corroborated these findings, identifying 440 DAMs, among which phospholipids, fatty acids, carboxylic acids, and amino acids were the most significantly altered. Lipid metabolism was the most severely affected module. Transcriptomic downregulation of genes involved in fatty acid and glycerophospholipid biosynthesis, coupled with dramatic changes in phospholipid and fatty acid levels, strongly indicates disrupted membrane lipid homeostasis. This dual evidence demonstrates that carvacrol primarily targets the cell membrane, increasing permeability and impairing structural integrity.

Transcriptomic suppression of oxidative phosphorylation and TCA cycle-related genes, together with metabolomic reductions in TCA intermediates and widespread perturbation of purine and nucleotide metabolism, reflect mitochondrial dysfunction and ATP depletion. For *P. manshurica*, carbon dioxide (the primary gas metabolite causing package swelling) originates from two dominant routes: glycolysis-driven sugar fermentation and aerobic TCA cycle respiration, while arginine catabolism serves as an auxiliary CO_2_-producing branch. All three gas-generating pathways require sufficient intracellular ATP to support sugar transmembrane transport, glycolytic enzyme activity, and intermediate metabolite turnover. The simultaneous blockage of the TCA cycle and oxidative phosphorylation induces systemic energy exhaustion, which directly stalls glycolysis—the primary source of fermentative CO_2_. Critically, impaired mitochondrial ATP synthesis eliminates the metabolic capacity to trigger compensatory carbon flux redistribution; even with TCA cycle interruption, the yeast cannot redirect carbon substrates toward alternative gas-producing reactions, so overall CO_2_ accumulation decreases rather than increases. The combined inhibition of TCA cycling and ATP biogenesis constitutes the core metabolic bottleneck suppressing gas production.

Broad dysregulation of amino acid metabolism further amplifies the reduction of CO_2_ output via two distinct mechanisms linked to alanine, aspartate, and arginine. First, alanine and aspartate function as vital carbon skeleton connectors between amino acid pools and central carbon metabolism: alanine is readily deaminated into pyruvate, and aspartate undergoes transamination to form oxaloacetate, both key upstream intermediates feeding the TCA cycle. Disturbed homeostasis of alanine and aspartate restricts carbon skeleton replenishment for the TCA cycle, further attenuating respiratory CO_2_ generation. Second, arginine metabolism operates as an independent auxiliary gas-producing pathway through the arginine deiminase cascade, where hydrolysis of carbamoyl phosphate releases free CO_2_. Collectively, disrupted amino acid metabolism restricts central carbon substrate supply and eliminates a supplementary gas-producing pathway, compounding the inhibitory effect on gas formation triggered by energy failure. Altered expression of genes involved in amino acid biosynthesis and degradation, combined with significant changes in amino acid abundance, also impairs global protein synthesis and stress resistance, further destabilizing yeast metabolic homeostasis and proliferative capacity.

Collectively, transcriptomic and metabolomic data consistently demonstrate that carvacrol inhibits gas production in *P. manshurica* through a multi-target mechanism: disrupting membrane integrity, impairing mitochondrial energy metabolism, and disturbing amino acid metabolism. The high consistency between gene expression and metabolite profiles confirms the reliability of the multi-omics results and provides a solid molecular basis for understanding the anti-spoilage mechanism of carvacrol.

## 4. Discussion

*P. manshurica*-mediated gas spoilage severely compromises the quality and shelf life of fermented foods, causing package swelling, product deformation, and economic losses [29]. This study systematically demonstrates that carvacrol, a natural phenolic compound, strongly inhibits gas production by *P. manshurica* and elucidates the underlying molecular mechanism via integrated transcriptomics and metabolomics. The findings highlight carvacrol as a promising natural preservative for controlling gas-producing spoilage yeasts in fermented food systems.

Compared with synthetic preservatives, carvacrol offers advantages of natural origin, low toxicity, and consumer acceptance [30]. Carvacrol, the principal active constituent of essential oils extracted from aromatic plants (e.g., oregano and thyme), is a natural phenolic compound granted Generally Recognized as Safe (GRAS) status by the U.S. Food and Drug Administration (FDA), with widely acknowledged safety [16]. In our previous study, we found that carvacrol exhibited potent inhibitory activity against *P. manshurica* with an EC_50_ value of 0.28 mg/L and at a dosage of 0.05 g/kg, carvacrol did not have any adverse impact on the flavor of the chili sauce products [6]. However, challenges remain for industrial application, including potential flavor impact, stability in complex food matrices, and cost [31]. In the field of animal husbandry, carvacrol has been shown to reduce gas and methane production in rumen fermentation by regulating the microbial community and metabolic pathways. For example, Benchaar and Hassanat reported that carvacrol exhibited significant anti-methanogenic effects in vitro, and at 1000 mg/L concentration, reduced total gas production, volatile fatty acids, acetate, propionate, ammonia, and methane emissions by approximately 22%, indicating inhibition of microbial fermentation and feed digestion [32]. These studies, together with our findings, confirm that carvacrol has broad-spectrum inhibitory activity against gas-producing microorganisms in different systems, and its mechanism of action is related to disrupting cell membrane integrity, impairing energy metabolism, and interfering with microbial community structure. Future studies should evaluate carvacrol’s effects on food sensory properties, optimize delivery systems (e.g., microencapsulation) to enhance stability and reduce dosage, and explore synergistic combinations with other natural compounds to improve antimicrobial efficacy. Additionally, the impact of carvacrol on the natural microbial community of fermented foods requires further investigation to ensure product safety and quality. Consistent with previous antimicrobial studies, our results confirm that carvacrol exerts a potent, concentration-dependent inhibitory effect on *P. manshurica* both in vitro and in situ in chili sauce [6]. Notably, carvacrol not only suppressed yeast growth but also specifically impaired gas-producing metabolism, representing a previously unreported anti-spoilage action. This dual effect distinguishes carvacrol from conventional preservatives that merely inhibit microbial proliferation, offering a targeted strategy to address gas spoilage. Considering carvacrol’s broad-spectrum antimicrobial property, we also note a potential concern: long-term intake of foods containing free carvacrol may pose mild inhibitory effects on beneficial human gut flora and disturb intestinal microbial homeostasis. Nevertheless, the optimal working concentration (0.05 g/kg) determined in this study is well below the acceptable daily intake (ADI) of carvacrol for humans, which greatly reduces such risks. Additionally, adopting microencapsulation and controlled-release delivery systems can isolate carvacrol from the gastrointestinal environment to a certain extent, weakening its activity against intestinal probiotics while retaining its anti-spoilage performance in fermented foods. The in situ validation in chili sauce further confirms its practical efficacy in complex food matrices, supporting industrial applicability.

Transcriptomic analysis revealed that carvacrol significantly altered the expression of 374 genes in *P. manshurica*, predominantly associated with membrane function, mitochondrial activity, and core metabolism. GO enrichment highlighted the membrane and mitochondrion as primary targets, consistent with the well-established mechanism of carvacrol in disrupting microbial membrane integrity and impairing mitochondrial function [16]. Disruption of membrane-related genes may increase membrane permeability, leading to intracellular leakage and impaired nutrient transport. Meanwhile, downregulation of mitochondrial and energy metabolism genes, including those involved in oxidative phosphorylation and the TCA cycle, directly compromises ATP production, which is essential for yeast growth and gas formation. Metabolomic analysis further corroborated the transcriptomic findings, identifying 440 DAMs enriched in fatty acid metabolism, sphingolipid metabolism, and oxidative phosphorylation. Carvacrol-induced reductions in TCA cycle intermediates and ATP directly link mitochondrial dysfunction to impaired energy supply. Altered fatty acid and phospholipid profiles reflect disrupted membrane lipid homeostasis, consistent with transcriptomic evidence of membrane damage. Additionally, changes in amino acid metabolism suggest impaired protein synthesis and stress adaptation, further suppressing yeast growth and metabolic activity.

Integrated transcriptome–metabolome analysis revealed a coordinated response: carvacrol disrupts membrane integrity, blocks energy metabolism via inhibition of the TCA cycle and oxidative phosphorylation, and interferes with amino acid metabolism, ultimately inhibiting gas production by *P. manshurica*. This multi-target mechanism provides a comprehensive molecular explanation for the observed phenotypic inhibition. This study clarifies the molecular mechanism by which carvacrol inhibits gas production by *P. manshurica* through multi-omics integration. The findings provide a theoretical basis for the application of carvacrol as a natural preservative in fermented foods and offer new insights into the development of targeted strategies for controlling gas-producing spoilage yeasts.

## 5. Conclusions

In summary, this study systematically demonstrated that carvacrol exerts a strong and concentration-dependent inhibitory effect on gas production by *P. manshurica* both in vitro and in situ in chili sauce. Through integrated transcriptomic and metabolomic analyses, we revealed the molecular mechanism underlying this inhibition: carvacrol disrupts cell membrane integrity, impairs mitochondrial function, blocks the tricarboxylic acid cycle and oxidative phosphorylation, and interferes with energy, lipid, and amino acid metabolism in *P. manshurica*, thereby suppressing its growth and gas production. These findings provide clear molecular evidence for the anti-spoilage activity of carvacrol and establish a theoretical basis for its application as a natural, safe, and effective preservative in fermented foods. The results of this study clarify the molecular mechanism by which carvacrol inhibits gas production in *P. manshurica* and provide a theoretical basis and technical support for the development and application of carvacrol as a natural and safe preservative in the fermented food industry.

## Figures and Tables

**Table 1:** Effect of different carvacrol concentrations on gas production by *P. manshurica* in vitro and in situ.

Condition	Concentration (mg/L)	Gas Production Volume (mL) ^1^
In vitro	0	1.3 ± 0.2
	0.25	0.8 ± 0.1
	0.50	0.4 ± 0.1
	0.75	0
	1.00	0
	1.25	0
In situ	0	0.5 ± 0.2
	0.05	0.2 ± 0.1
	0.10	0

^1^ mean ± standard deviation (SD), n = 3.

## Data Availability

The original contributions presented in this study are included in the article/Supplementary Material. Further inquiries can be directed to the corresponding authors.

## Supplementary Materials

The following supporting information can be downloaded at: https://www.mdpi.com/article/10.3390/microorganisms14081615/s1, Table S1: GO enrichment analysis of DEGs; Table S2: KEGG pathway enrichment analysis of DEGs; Table S3: DAMs of *P. manshurica* under carvacrol stress; Table S4: Superclass classification of identified metabolites; Table S5: Compound classification of DAMs; Table S6: Pathway classification of DAMs; Table S7: VIP values analysis of DAMs; Table S8: KEGG pathway enrichment analysis of DAMs.
